# Cost‐effectiveness of multiparametric magnetic resonance imaging and MRI‐guided biopsy in a population‐based prostate cancer screening setting using a micro‐simulation model

**DOI:** 10.1002/cam4.3932

**Published:** 2021-05-15

**Authors:** Abraham M. Getaneh, Eveline AM Heijnsdijk, Harry J. de Koning

**Affiliations:** ^1^ Department of Public Health Erasmus MC University Medical Center Rotterdam Rotterdam the Netherlands

**Keywords:** Cost‐effectiveness analysis, mpMRI, MRI‐guided biopsy, prostate cancer, PSA Screening

## Abstract

**Background:**

The introduction of multiparametric magnetic resonance imaging **(**mpMRI) and MRI‐guided biopsy has improved the diagnosis of prostate cancer. However, it remains uncertain whether it is cost‐effective, especially in a population‐based screening strategy.

**Methods:**

We used a micro‐simulation model to assess the cost‐effectiveness of an MRI‐based prostate cancer screening in comparison to the classical prostate‐specific antigen (PSA) screening, at a population level. The test sensitivity parameters for the mpMRI and MRI‐guided biopsy, grade misclassification rates, utility estimates, and the unit costs of different interventions were obtained from literature. We assumed the same screening attendance rate and biopsy compliance rate for both strategies. A probabilistic sensitivity analysis, consisting of 1000 model runs, was performed to estimate a mean incremental cost‐effectiveness ratio (ICER) and assess uncertainty. A €20,000 willingness‐to‐pay (WTP) threshold per quality‐adjusted life year (QALY) gained, and a discounting rate of 3.5% was considered in the analysis.

**Results:**

The MRI‐based screening improved the life‐years (LY) and QALYs gained by 3.5 and 3, respectively, in comparison to the classical screening pathway. Based on the probabilistic sensitivity analyses, the MRI screening pathway leads to total discounted mean incremental costs of €15,413 (95% confidence interval (CI) of €14,556–€16,272) compared to the classical screening pathway. The corresponding discounted mean incremental QALYs gained was 1.36 (95% CI of 1.31–1.40), resulting in a mean ICER of €11,355 per QALY gained. At a WTP threshold of €20,000, the MRI screening pathway has about 84% chance to be more cost‐effective than the classical screening pathway.

**Conclusions:**

For triennial screening from age 55–64, incorporation of mpMRI as a reflex test after a positive PSA test result with a subsequent MRI‐guided biopsy has a high probability to be more cost‐effective as compared with the classical prostate cancer screening pathway.

## Graphical abstract

Incorporation of mpMRI as a triage test before biopsy followed by MRI‐guided biopsy improved the life‐years (LY) and QALYs gained, and has a high probability to be more cost‐effective as compared with the regular prostate cancer screening pathway.

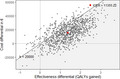

## 1 BACKGROUND

Despite the presence of compelling evidence regarding the beneficial effects of prostate‐specific antigen (PSA) screening from a trial and modeling studies,^1^, ^2^, ^3^ almost no country implemented PSA screening at a population level.^4^ This is mainly due to the fact that PSA screening is associated with high risk of overdiagnosis and overtreatment. However, the European Urology of Association (EAU) recently stated that the European union can no longer overlook prostate cancer, and the introduction of PSA screening at a European level needs to be rediscussed by taking into consideration the current evidences about prostate cancer screening.^5^ A recent brief correspondence to the European Association of Urology (EAU) emphasized the importance of introducing organized PSA screening at a population level in order to reduce mortality from prostates cancer.^6^ The authors indicated that multiparametric magnetic resonance (mpMRI) should be used as a reflex test after a positive PSA test result to select men for biopsy.

The introduction of mpMRI and targeted biopsy has improved the diagnosis of prostate cancer. Several studies reported that the use of mpMR as a triage before biopsy and followed by MRI‐guided biopsy can substantially reduce the detection of low‐grade prostate cancers and also result in a better detection of clinically significant cancers compared to the classical screening with an upfront transrectal ultrasound‐guided biopsy (TRUSGB) for all men with a positive PSA test result.^7^, ^8^, ^9^, ^10^, ^11^ While the benefits of using mpMRI with a subsequent MRI‐targeted biopsy have become more clear, its cost‐effectiveness remains uncertain, especially for a screening strategy at a population level.

Although some studies reported the cost‐effectiveness of mpMRI and subsequent targeted biopsy,^12^, ^13^, ^14^, ^15^ to our knowledge, no study has yet quantified the cost‐effectiveness in a population‐based screening strategy, particularly in the European situation. Screening at a population level should have a clear starting and stopping age of screening and intervals to screen. A study by Barnett *et al*.^16^ that modeled screening from 55–69 at 2 years intervals reported the cost‐effectiveness of mpMRI and targeted fusion biopsy. However, the setting is in the USA, where the costs of MRI are much different from the costs in Europe. The aim of this study was to investigate the cost‐effectiveness of MRI‐based prostate cancer screening pathway compared to the classical screening pathway at a population level, using a base model which was calibrated to the European Randomized Study of Screening for Prostate Cancer (ERSPC) data and Dutch prostate cancer incidence and mortality data.^17^ In this study, the MRI screening pathway represents a positive PSA test (≥3 ng/ml) followed by mpMRI test and MRI‐guided biopsy (for those men positive on mpMRI test), whereas the classical screening pathway refers to a positive PSA test (≥3 ng/ml) followed by TRUSGB.

## 2 MATERIALS AND METHODS

### 2.1 Model overview

In the present study, the micro‐simulation screening analysis (MISCAN) prostate cancer model was used.^3^, ^18^, ^19^ Taking variation into account, the model simulates life histories for each individual starting from birth to death. Everyone in the simulation starts with no prostate cancer. Once a malignant prostate tumor initiated in any individual in the model, the progression of the cancer is simulated as a sequence of preclinical and clinical states. In combination with three stages (T1, T2, and T3), three Gleason scores (7, less than 7, and greater than 7), and three metastatic states (local‐regional and distant), the model has 18 preclinical states. There is also a chance for the tumor to progress from each preclinical state to the next T‐stage, or change to a higher Gleason score, or it may be clinically diagnosed (Figure S1). Furthermore, the tumor has a chance to metastasize from a local‐regional state into a distant state. For every individual, two life histories are projected by the model: one without screening and the other with screening. A screen‐detected cancer that would not lead to a clinical diagnosis in case of no screening is considered as an overdiagnosed cancer.^11^

Using Surveillance, Epidemiology, and End Results (SEER) data (1983–1986), baseline prostate cancer survival (without screening and localized treatment) in the model was determined at clinical diagnosis.^20^ In order to model death other than prostate cancer, we used a life table of Dutch population.^21^ To model the effects of treatment on localized prostate cancer, a 0.56 relative risk of dying was assumed for radical prostatectomy (RP) as compared to watchful waiting.^22^ We assumed the same treatment benefit for radiation therapy (RP). The distributions of treatments were based on age, stage, and Gleason score.^2^, ^23^ The benefit of PSA screening on prostate cancer mortality was simulated as a function on lead time based on a lead time‐dependent cure probability.^2^ The years by which cancer detection using screening precede clinical detection is termed as a lead time.^11^ Detailed information about the model including calibration and validation can be found on literature^3^, ^17^, ^18^ and using: https://cisnet.flexkb.net/mp/pub/cisnet_modelprofile_prostate_erasmus_001_12152009_69754.pdf

### 2.2 Screening protocol

The screening intervals, start and end age in the present study was based on the optimal screening strategy reported in a cost‐effectiveness analyses using the same base model, which is from age 55–64 at 3 years interval with an 80% screening attendance.^17^ A 90% biopsy compliance rate with a biopsy sensitivity 90% was assumed based on the ERSPC Rotterdam data.^24^, ^25^ We kept this screening protocol for the classical screening pathway of the current study. For the MR screening pathway, we added mpMRI as triage test between a positive PSA test and biopsy. This means, men after a positive PSA test were further selected using an mpMRI test before biopsy, and only those men positive at mpMRI (PIRADS scores of 3–5) went to biopsy. Furthermore, for the MRI screening pathway, we replaced the TRUSGB with MRI‐guided biopsy (Figure S2). The screening attendance rate and biopsy compliance rate that we used in the MRI screening pathway are the same as in the classical screening pathway. The test sensitivity parameters for the mpMRI and MRI‐guided biopsy were obtained from literature, mainly meta‐analyses (Table 1). Misclassification of grades (misclassifying a clinically significant cancer into an insignificant cancer at biopsy) was also included in the model both for the MRI‐guided biopsy and TRUSGB. We used an 8.7% misclassification rate for the MRI‐guided biopsy.^8^ For the TRUSGB biopsy, we obtained different values from literature,^8^, ^26^, ^27^ and used the intermediate 36.3% (16.8%–60%).

**TABLE 1:** The test sensitivity values for the MRI pathway, the utility values and durations of the health states, and the unit costs of interventions

Parameters included in the probabilistic sensitivity analyses		
Variables	Values	Sources
Sensitivity of mpMRI for HGC^a^	0.94 (SD: 0.06)^d^	Sathianathen et al 2019^38^
Overall sensitivity of mpMRI^b^	0.74 (SD: 0.06)^d^	de Rooij et al. 2014^39^
Sensitivity of MRI‐guided biopsy for HGC	0.91 (SD: 0.05)^d^	Schoots et al. 2015^34^
Sensitivity of MRI‐guided biopsy for LGC^c^	0.44 (SD:0.05)^d^	Schoots et al. 2015^34^
Unit costs of mpMRI	€345 (min = €293, max = €397)^e^	de Rooij et al. 2014^13^
Unit costs of MRIGB	€800 (min = €680, max = 920)^e^	de Rooij et al. 2014^13^
Unit costs of TRUSGB	€247 (min = €210, max = €284)^e^	Heijnsdijk etal 2015^3^

Remaining unit costs in Euro used in the model (common for both strategies)		
Variables	Values	Heijnsdijk et al 2015^3^
PSA screening	35	
Staging	290	
Radical prostatectomy (RP)	17,119	
Radiation therapy (RT)	20,568	
Active surveillance (AS)	2,303	
Follow‐up	218	
Advanced disease (Palliative treatment)	17,800	

Utility values and duration of health states used in the model Heijnsdijk et al 2012^18^ (common for both strategies, except biopsy and mpMRI)		
Health states	Utility estimates (Favorable, Unfavorable)	Duration
PSA screening attendance	0.99 (1, 0.99)	1 week
mpMRI	0.96^40^	1 week
TRUSGB	0.90 (0.94, 0.87)	3 weeks
MRIGB^f^	0.95 (0.97, 0. 93)	3 weeks
Diagnosis	0.80 (0.85, 0.75)	1 month
RP	0.67 (0.90, 0.56)	2 months
RT	0.73 (0.91, 0.71)	2 months
AS	0.97 (1, 0.85)	7 years
2 months to 1 year RP	0.77 (0.91, 0.70)	10 months
2 months to 1 year RT	0.78 (0.88, 0.61)	10 months
Postrecovery period	0.95 (1, 0.93)	9 years
Palliative therapy	0.60 (0.24, 0.86)	30 months
Terminal illness	0.40 (0.24, 0.56)	6 months

Abbreviations: max, maximum; min, minimum; mpMRI, multiparametric magnetic resonance imaging; MRI, magnetic resonance imaging; TRUSGB, transrectal ultrasound‐guided biopsy.

^a^ HGC=high‐grade cancer

^b^ Assumed as a sensitivity of mpMRI for LGC.

^c^ LGC=low‐grade cancer

^d^ The standard deviations are based on de Rooij et al. 2014^13^

^e^ The base value is varied by ±15% for the max and min

^f^ Because usually less biopsy complications are associated with MRIGB than TRUSGB, we assumed a 50% lower utility loss due to MRIGB than TRUSGB.

### 2.3 Costs

All the unit costs included in this study were obtained from literature and reported in Euros (Table 1). The number of screening visits, positive biopsies, diagnoses, treatments, and life years were estimated by the model. In order to determine the number of negative biopsies, we calculated the total number of biopsies based on detected cancers and a positive predictive value of a biopsy as described on literature.^11^, ^17^ Indirect costs were not included in this study. A 3.5% discounting rate was used for both costs and effects.

### 2.4 Utilities and quality of life

Most of the utility values and duration of health states were obtained from literature,^18^ (Table 1). The loss in utility was calculated by subtracting the utility value from 1. The product of the number of men in a given health state with the loss in utility and duration of the health state gives the loss in quality of life.^17^

### 2.5 Analysis

For both strategies, the undiscounted LY gained, QALYs gained, and the number of men biopsied were from a single model run. The net effects and costs in each strategy were compared with a no screening strategy. The mean discounted total net costs of screening, diagnosis, and treatment, and palliative care, the mean discounted net QALYs gained and the mean total incremental net costs along with 95% CI, the mean incremental cost‐effectiveness ratio (ICER), and the mean incremental net monetary benefit (iNMB) were based on probabilistic sensitivity analyses. To estimate the ICER, we divided the difference in total net costs between the MRI screening pathway and the classical screening pathway by the difference in net QALYs gained between the two strategies. A willingness‐to‐pay threshold (WTP) of €20,000, which is a common Dutch WTP,^28^ was used to determine the cost‐effective of a given strategy. If the ICER of a given strategy is lower than this WTP, it is cost effective. The mean iNMB was calculated by multiplying the incremental net effects (QALYs) with a WTP (€20,000) and subtracting the incremental net costs.

In the probabilistic sensitivity analyses, we performed 1,000 simulations in which selected model parameters were varied (based on distribution) simultaneously. A large sample size (10 million men) was used in each simulation, which eliminated stochastic noise in the model. The parameters included in the probabilistic sensitivity analyses were mainly those parameters which are not common in the two strategies. This includes the test sensitivity values of mpMRI, sensitivity values of MRI‐guided biopsy, costs of mpMRI, costs of MRI‐ guided biopsy, and costs of TRUSGB. The test sensitivity values were varied using their base value and standard deviation. For the costs, we used a Pert distribution with the most likely (base) value and an assumption of ±15% for the minimum and maximum values (Table 1). The uncertainty around utility values and remaining costs were tested only using a one‐way sensitivity analysis (because of labor constraints). The baseline utility values were varied using their favorable and unfavorable estimates, obtained from literature,^18^ and the costs were varied by ±15%.

For postprocessing of the outputs, we used R software together with the Bayesian cost‐effectiveness analysis (BCEA) and ggplot2 packages to obtain the cost‐effectiveness plain with mean ICER and the cost acceptability curves.^29^ We used Rmisc package^30^ to obtain the mean incremental net costs and effects with their 95% CI based on the 1,000 model runs for the probabilistic sensitivity analysis.

## 3 RESULTS

### 3.1 Undiscounted effects from the base model

For triennial screening from age 55–64, the MRI screening pathway resulted in additional 3.5 life‐years gained and 3 additional QALYs gained per 1,000 men invited to screening and followed over their lifetime period. Furthermore, the number of biopsied men reduced by 30% when the MRI screening pathway was used (Table 2).

**TABLE 2:** Estimated life time screening outcomes and results of probabilistic sensitivity analysis per 1000 men invited

	Classical pathway (C)	MRI pathway (M)	Difference (M‐C)
Screening outcomes from single run			
Number biopsied	396	278	−118 (30%)
Life years gained*	81.5	85	+3.5 (4%)
Quality‐adjusted life years gained*	77.2	80.2	+3.0 (4%)
PS analysis outcome, 3.5% discounted			
Mean net costs (in €) of *			
Screening	80,118	156,429	+76,311 (49%)
Diagnosis and treatment	317,999	258,206	−59,793 (19%)
Palliative care	−60,145	−61,250	−1,105 (2%)
Mean total net costs	337,972	353,385	+15,413 (4.4%)
Mean QALY gained*	24.09	25.45	+1.36 (5.3%)
Mean incremental total net costs with 95% CI in the bracket	—	15,413 (14,556; 16,272)	+15,413 (14,556; 16,272)
Mean incremental QALYs gained with 95% CI in the bracket	—	1.36 (1.31, 1.40)	+ 1.36 (1.31, 1.40)
Mean ICER	—	11,355	+11,355
Mean incremental net monetary benefit (iNMB)	—	11,735	+11,735

Abbreviations: CI, confidence interval, M, MRI pathway, PS analysis, Probabilistic sensitivity analysis, C, classical pathway.

* Compared to no screening

### 3.2 Probabilistic sensitivity analysis

**FIGURE 1:**
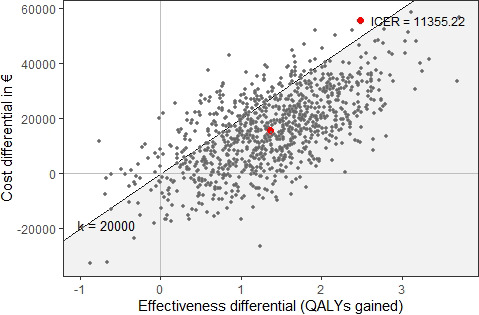
Cost‐effectiveness plain of the MRI screening pathway versus the classical pathway at a WTP threshold of €20,000. In the northeast quadrant, the MRI screening pathway is more effective and more costly; in the southeast quadrant, it is more effective and less costly (dominant); in the northwest quadrant, it is less effective and more costly (dominated); and in the southwest quadrant, it is less effective and less costly than the classical screening pathway

The results show that the mean discounted incremental costs of screening, diagnosis and treatment, and palliative care of the MRI screening pathway versus the classical pathway were €76,300, €‐59,793, and €‐1,105, respectively, resulting in a total mean incremental costs of €15,413 (95% confidence interval of €14,556–€16,272) for 1,000 men invited. The associated discounted mean incremental QALYs gained was 1.36, with a 95% confidence interval of 1.31–1.40 (Table 2). The mean ICER of the MRI screening pathway versus classical screening pathway was €11,355 (Table 2 and Figure 1). The cost‐effectiveness plane (Figure 1) shows the uncertainty around the mean ICER estimate, and the majority of the incremental net cost‐effect pairs gathered in the northeastern part of the plane below the WTP threshold line. In the northeast part of the plane, the MRI strategy is more effective and more expensive. The probabilities that the incremental cost‐effect pairs of the MRI pathway, compared to the classical screening pathway, to fall in northeast and southeast quadrants were 85.2% and 11.3%, respectively (Figure S3).

**FIGURE 2:**
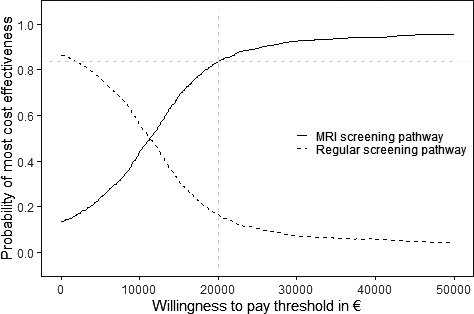
Cost‐effectiveness acceptability curves for the MRI screening pathway and classical (regular) pathway

The cost‐effectiveness acceptability curves at Figure 2 show that the MRI screening pathway had a high probability of being more cost‐effective (84%) compared with the classical screening pathway, using a €20,000 WTP threshold per QALY gained. At this WTP threshold, the MRI screening pathway has also a positive mean iNMB of €11,735 compared with the classical screening pathway (Table 2).

The one‐way sensitivity analysis did not change the ICER substantially, ranging only between €10,000 and €13,700 (Table S1). Although the change is not substantial, the cost‐effectiveness became better for the MRI‐based screening strategy when the utility estimates for biopsy, diagnosis, treatments, palliative care, and advanced disease were unfavorable. Similarly, the ICER decreased when the costs of staging, treatment, and advanced disease care increased.

## 4 DISCUSSION

The results from the model that accounts for long‐term prediction of costs and effects suggest that the use of MRI screening pathway is more cost‐effective than the classical prostate cancer screening pathway. The MRI pathway reduced the diagnosis and treatment costs by 19% and that of palliative care by 2% in comparison to the classical pathway. This reduction in diagnosis and treatment costs is mainly due to the lower sensitivity of mpMRI and MRI‐guided biopsy for low‐grade prostate cancer that reduces unnecessary biopsy and treatment. Generally, mpMRI and MRI‐guided biopsy have lower sensitivity for low‐grade cancer and higher sensitivity for clinically significant cancer ^31^, ^32^, ^33^ than the traditional random biopsy (TRUSGB).^34^ The latter can explain the reduction in the costs of the palliative care reported in the current study which in turn reduces the occurrence of advanced prostate cancer (prostate cancer with clinical symptoms). In comparison to the classical screening pathway, the MRI pathway also resulted in additional LY gained and QALYs gained. Reduction in biopsy procedure, overlooking of low‐grade cancer, and better detection of clinically significant cancer ^34^, ^35^ due to the MRI pathway could explain these findings. Whether the MRI screening strategy is cost‐effective than the classical screening pathway depends on the WTP threshold, and according to our results at €20,000 cut‐off, the MRI screening pathway is most cost‐effective in the majority of the model runs (84%) done for the probabilistic sensitivity analyses. The reduction in biopsy costs due to avoiding unnecessary biopsies, treatment costs due to avoiding overtreatment, and the reduction in palliative cares costs due to improved detection of clinically significant cancers, as well as the modest increment of the QALYs gained in the MRI screening pathway explain how this strategy leads to a high probability to be cost‐effective as compared to the regular screening pathway.

Although their screening strategies differed, some published studies showed that the use of mpMRI and MRI‐guided biopsy is cost‐effective,^12^, ^13^ which is in agreement with our findings. A cost‐effectiveness analysis from the USA reported a higher ICER than the current finding,^16^ and this could be mainly because of the costs of MRI in the USA are much higher than the costs in Europe that we used in this study. It should be noted that the results may not be directly comparable with the present study due to several reasons (such as screening strategies, model performance, data used, and follow‐up period), but the general conclusions are consistent. The 30% reduction in biopsy procedure due to the MRI screening pathway in this study is consistent with a recent MRI study.^10^

Major strength of the present study is that we determined the cost‐effectiveness of the MRI screening pathway at population level which was not reported before, particularly in the European situation. Another strength of the present study is that the MISCAN prostate model, we used in this study, includes the unobservable prostate cancer natural history, and also allows us to estimate effects of screening over life time periods, which is unlikely in trial studies, and most of other modeling cost‐effectiveness studies.^12^, ^13^, ^15^

This study is also subjected to certain limitations. First, we did not account costs of biopsy complications. There is more risks of complication and subsequent increment of health care costs due to TRUSGB biopsy than MRI‐guided biopsy.^36^, ^37^ Therefore, the cost‐effectiveness would be even more in favor of the MRI screening pathway if these costs were included. Second, assumptions were made for certain model parameters when data are not available. Another limitation of the present study is that treatment options were assumed to be the same and will not change in both strategies. However, how diagnosed cancer should be treated may depend on the MRI outcome, and also treatment behavior may alter in time. More studies are needed to assess whether it is effective to make treatment decisions based on MRI test results.

In conclusion, our study suggests that for triennial screening from age 55 to 64, incorporating mpMRI as a triage test in prostate cancer screening before biopsy with subsequent MRI‐guided biopsy has a high probability to be more cost‐effective than the classical screening pathway.

## 5 ETHICS APPROVAL

Not applicable (No human or animal subjects were involved in this study. It is a modeling study).

## CONFLICT OF INTEREST

None.

## AUTHOR CONTRIBUTIONS

Conceptualization: all authors. Data curation: A.M.G. and E.A.M.H. Formal analysis: A.M.G. Funding acquisition: H.J.K. Investigation: all authors. Methodology: all authors. Writing—original draft: A.M.G. Writing—review and editing: all authors. All authors have read and approved the manuscript.

## Supporting information

Figure S1

Figure S2

Figure S3

Table S1

## Data Availability

All data included in the manuscript.

## References

[1] Hugosson J , Roobol MJ , Månsson M , et al. A 16‐yr Follow‐up of the European randomized study of screening for prostate cancer. Eur Urol. 2019;76(1):43‐51. 30824296 10.1016/j.eururo.2019.02.009 PMC7513694

[2] de Koning HJ , Gulati R , Moss SM , et al. The efficacy of prostate‐specific antigen screening: Impact of key components in the ERSPC and PLCO trials. Cancer. 2018;124(6):1197‐1206. 29211316 10.1002/cncr.31178 PMC5839977

[3] Heijnsdijk EAM , De Carvalho TM , Auvinen A , et al. Cost‐effectiveness of prostate cancer screening: a simulation study based on ERSPC data. JNCI: Journal of the National Cancer Institute. 2015;107(1):366–374. 25505238 10.1093/jnci/dju366 PMC4296196

[4] Shieh Y , Eklund M , Sawaya GF , Black WC , Kramer BS , Esserman LJ . Population‐based screening for cancer: hope and hype. Nature reviews Clinical oncology. 2016;13(9):550‐565. 10.1038/nrclinonc.2016.50 PMC6585415 27071351

[5] European . Policy paper on PSA screening for prostate cancer: Has the time come to reconsider structured population‐based PSA screening for prostate cancer? *European Association of Urology*. https://www.newsbook.com.mt/wp‐content/uploads/2019/04/EAU_policy‐briefing_PSA.pdf.

[6] Gandaglia G , Albers P , Abrahamsson P‐A , et al. Structured population‐based prostate‐specific antigen screening for prostate cancer: the European association of urology position in 2019. Eur Urol. 2019;76(2):142‐150. 31092338 10.1016/j.eururo.2019.04.033

[7] Ahmed HU , Bosaily AE‐S , Brown LC , et al. Diagnostic accuracy of multi‐parametric MRI and TRUS biopsy in prostate cancer (PROMIS): a paired validating confirmatory study. Lancet. 2017;389(10071):815‐822. 28110982 10.1016/S0140-6736(16)32401-1

[8] Ahdoot M , Wilbur AR , Reese SE , et al. MRI‐targeted, systematic, and combined biopsy for prostate cancer diagnosis. N Engl J Med. 2020;382(10):917‐928. 32130814 10.1056/NEJMoa1910038 PMC7323919

[9] Pokorny MR , De Rooij M , Duncan E , et al. Prospective study of diagnostic accuracy comparing prostate cancer detection by transrectal ultrasound–guided biopsy versus magnetic resonance (MR) imaging with subsequent MR‐guided biopsy in men without previous prostate biopsies. Eur Urol. 2014;66(1):22‐29. 24666839 10.1016/j.eururo.2014.03.002

[10] Kasivisvanathan V , Rannikko AS , Borghi M , et al. MRI‐targeted or standard biopsy for prostate‐cancer diagnosis. N Engl J Med. 2018;378(19):1767‐1777. 29552975 10.1056/NEJMoa1801993 PMC9084630

[11] Getaneh AM , Heijnsdijk EAM , de Koning HJ . The comparative effectiveness of mpMRI and MRI‐guided biopsy vs regular biopsy in a population‐based PSA testing: a modeling study. Sci Rep. 2021;11(1):1‐8. 33469144 10.1038/s41598-021-81459-2 PMC7815791

[12] Pahwa S , Schiltz NK , Ponsky LE , Lu Z , Griswold MA , Gulani V . Cost‐effectiveness of MR imaging–guided strategies for detection of prostate cancer in biopsy‐naive men. Radiology. 2017;285(1):157‐166. 28514203 10.1148/radiol.2017162181 PMC5621719

[13] de Rooij M , Crienen S , Witjes JA , Barentsz JO , Rovers MM , Grutters JPC . Cost‐effectiveness of magnetic resonance (MR) imaging and MR‐guided targeted biopsy versus systematic transrectal ultrasound–guided biopsy in diagnosing prostate cancer: a modelling study from a health care perspective. Eur Urol. 2014;66(3):430‐436. 24377803 10.1016/j.eururo.2013.12.012

[14] Cerantola Y , Dragomir A , Tanguay S , Bladou F , Aprikian A , Kassouf W . Cost‐effectiveness of multiparametric magnetic resonance imaging and targeted biopsy in diagnosing prostate cancer. Urol Oncol. 2016;34(3):119.e1‐119.e9. 10.1016/j.urolonc.2015.09.010 26602178

[15] Venderink W , Govers TM , de Rooij M , Fütterer JJ , Sedelaar JPM . Cost‐effectiveness comparison of imaging‐guided prostate biopsy techniques: systematic transrectal ultrasound, direct in‐bore MRI, and image fusion. Am J Roentgenol. 2017;208(5):1058‐1063. 28225639 10.2214/AJR.16.17322

[16] Barnett CL , Davenport MS , Montgomery JS , Wei JT , Montie JE , Denton BT . Cost‐effectiveness of magnetic resonance imaging and targeted fusion biopsy for early detection of prostate cancer. BJU Int. 2018;122(1):50‐58. 10.1111/bju.14151 29388388

[17] Getaneh AM , Heijnsdijk EAM , Roobol MJ , de Koning HJ . Assessment of harms, benefits, and cost‐effectiveness of prostate cancer screening: A micro‐simulation study of 230 scenarios. Cancer Med. 2020;19:e157. 10.1002/cam4.3395 PMC7571827 32813910

[18] Heijnsdijk EAM , Wever EM , Auvinen A , et al. Quality‐of‐life effects of prostate‐specific antigen screening. N Engl J Med. 2012;367(7):595‐605. 22894572 10.1056/NEJMoa1201637 PMC4982868

[19] Wever EM , Draisma G , Heijnsdijk EAM , et al. Prostate‐specific antigen screening in the United States vs in the European randomized study of screening for prostate cancer‐rotterdam. J Natl Cancer Inst. 2010;102(5):352‐355. 20142584 10.1093/jnci/djp533 PMC2831048

[20] Heijnsdijk EAM , Gulati R , Tsodikov A , et al. Lifetime benefits and harms of PSA‐based risk screening for prostate cancer. JNCI: Journal of the National Cancer Institute. 2020:1013–1020. 32067047 10.1093/jnci/djaa001 PMC7566340

[21] van der Meulen A . Life tables and Survival analysys. Netherlands: Statstics; 2012.

[22] Bill‐Axelson A , Holmberg L , Garmo H , et al. Radical prostatectomy or watchful waiting in early prostate cancer. N Engl J Med. 2014;370(10):932‐942. 24597866 10.1056/NEJMoa1311593 PMC4118145

[23] Etzioni R , Gulati R , Tsodikov A , et al. The prostate cancer conundrum revisited: treatment changes and prostate cancer mortality declines. Cancer. 2012;118(23):5955‐5963. 22605665 10.1002/cncr.27594 PMC3424303

[24] Schröder FH , van den Bergh RCN , Wolters T , et al. Eleven‐year outcome of patients with prostate cancers diagnosed during screening after initial negative sextant biopsies. Eur Urol. 2010;57(2):256‐266. 19913350 10.1016/j.eururo.2009.10.031

[25] Postma R , Schröder FH , van Leenders GJLH , et al. Cancer detection and cancer characteristics in the European randomized study of screening for prostate cancer (ERSPC)–section rotterdam: a comparison of two rounds of screening. Eur Urol. 2007;52(1):89‐97. 17257742 10.1016/j.eururo.2007.01.030

[26] Epstein JI , Feng Z , Trock BJ , Pierorazio PM . Upgrading and downgrading of prostate cancer from biopsy to radical prostatectomy: incidence and predictive factors using the modified Gleason grading system and factoring in tertiary grades. Eur Urol. 2012;61(5):1019‐1024. 22336380 10.1016/j.eururo.2012.01.050 PMC4659370

[27] Beckmann K , O'Callaghan M , Vincent A , et al. Extent and predictors of grade upgrading and downgrading in an Australian cohort according to the new prostate cancer grade groupings. Asian J Urol. 2019;6(4):321‐329. 31768317 10.1016/j.ajur.2019.03.001 PMC6872773

[28] Zwaap J , Knies S , Van der Meijden C , Staal P , Van der Heiden L . Kosteneffectiviteit in de praktijk. Diemen: Zorginstituut Nederland; 2015.

[29] Baio G , Berardi A , Heath A , Baio MG , Imports M, Package ‘BCEA’. 2019.

[30] Rmisc HRM . Ryan miscellaneous. R package version 1.5. CRAN. 2013.

[31] Venderink W , van Luijtelaar A , Bomers JGR , et al. Results of targeted biopsy in men with magnetic resonance imaging lesions classified equivocal, likely or highly likely to be clinically significant prostate cancer. Eur Urol. 2018;73(3):353‐360. 28258784 10.1016/j.eururo.2017.02.021

[32] Turkbey B , Brown AM , Sankineni S , Wood BJ , Pinto PA , Choyke PL . Multiparametric prostate magnetic resonance imaging in the evaluation of prostate cancer. CA Cancer J Clin. 2016;66(4):326‐336. 26594835 10.3322/caac.21333 PMC7511979

[33] Stabile A , Giganti F , Emberton M , Moore CM . MRI in prostate cancer diagnosis: do we need to add standard sampling? A review of the last 5 years. Prostate Cancer Prostatic Dis. 2018;21(4):473‐487. 30104656 10.1038/s41391-018-0071-8

[34] Schoots IG , Roobol MJ , Nieboer D , Bangma CH , Steyerberg EW , Hunink MGM . Magnetic resonance imaging–targeted biopsy may enhance the diagnostic accuracy of significant prostate cancer detection compared to standard transrectal ultrasound‐guided biopsy: a systematic review and meta‐analysis. Eur Urol. 2015;68(3):438‐450. 25480312 10.1016/j.eururo.2014.11.037

[35] Siddiqui MM , Rais‐Bahrami S , Turkbey B , et al. Comparison of MR/ultrasound fusion–guided biopsy with ultrasound‐guided biopsy for the diagnosis of prostate cancer. JAMA. 2015;313(4):390‐397. 25626035 10.1001/jama.2014.17942 PMC4572575

[36] Merriel SWD , Hardy V , Thompson MJ , Walter FM , Hamilton W . Patient‐centered outcomes from Multiparametric MRI and MRI‐guided biopsy for prostate cancer: a systematic review. J Am Coll Radiol. 2019;17(4):486–495. 31541653 10.1016/j.jacr.2019.08.031 PMC7132450

[37] Loeb S , Vellekoop A , Ahmed HU , et al. Systematic review of complications of prostate biopsy. Eur Urol. 2013;64(6):876‐892. 23787356 10.1016/j.eururo.2013.05.049

[38] Sathianathen NJ , Konety BR , Alarid‐Escudero F , Lawrentschuk N , Bolton DM , Kuntz KM . Cost‐effectiveness analysis of active surveillance strategies for men with low‐risk prostate cancer. Eur Urol. 2019;75(6):910‐917. 30425010 10.1016/j.eururo.2018.10.055

[39] de Rooij M , Hamoen EHJ , Fütterer JJ , Barentsz JO , Rovers MM . Accuracy of multiparametric MRI for prostate cancer detection: a meta‐analysis. Am J Roentgenol. 2014;202(2):343‐351. 24450675 10.2214/AJR.13.11046

[40] Grann VR , Patel PR , Jacobson JS , et al. Comparative effectiveness of screening and prevention strategies among BRCA1/2‐affected mutation carriers. Breast Cancer Res Treat. 2011;125(3):837‐847. 20644999 10.1007/s10549-010-1043-4 PMC3615889

