# Supplementary figures and images for: Cost‐effectiveness of multiparametric magnetic resonance imaging and MRI‐guided biopsy in a population‐based prostate cancer screening setting using a micro‐simulation model
Source: Cancer Med. 2021 May 15;10(12):4046–53. doi: 10.1002/cam4.3932 (PMC8209626; doi:10.1002/cam4.3932)

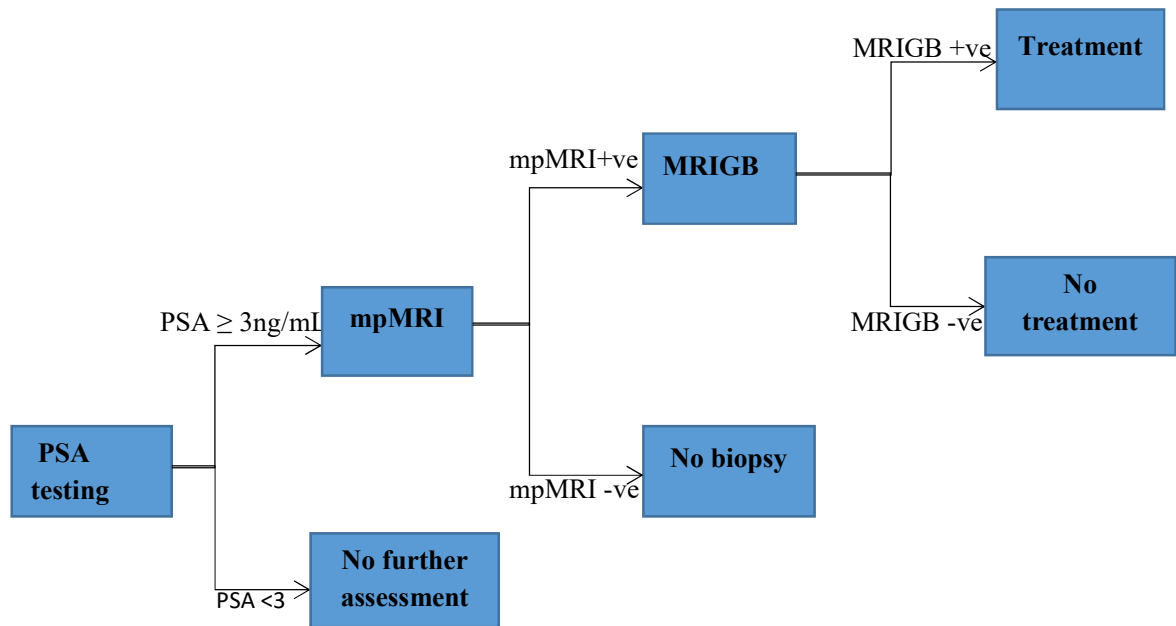

**Figure S2.** Schematic representation of the MRI pathway.

Supplement: Supplementary file 2 — Figure S2 [file CAM4-10-4046-s004.pdf]
