# Supplementary material for: Cost‐effectiveness of multiparametric magnetic resonance imaging and MRI‐guided biopsy in a population‐based prostate cancer screening setting using a micro‐simulation model
Source: Cancer Med. 2021 May 15;10(12):4046–53. doi: 10.1002/cam4.3932 (PMC8209626; doi:10.1002/cam4.3932)
Supplement: Supplementary file 3 — Figure S3 [file CAM4-10-4046-s001.pdf]

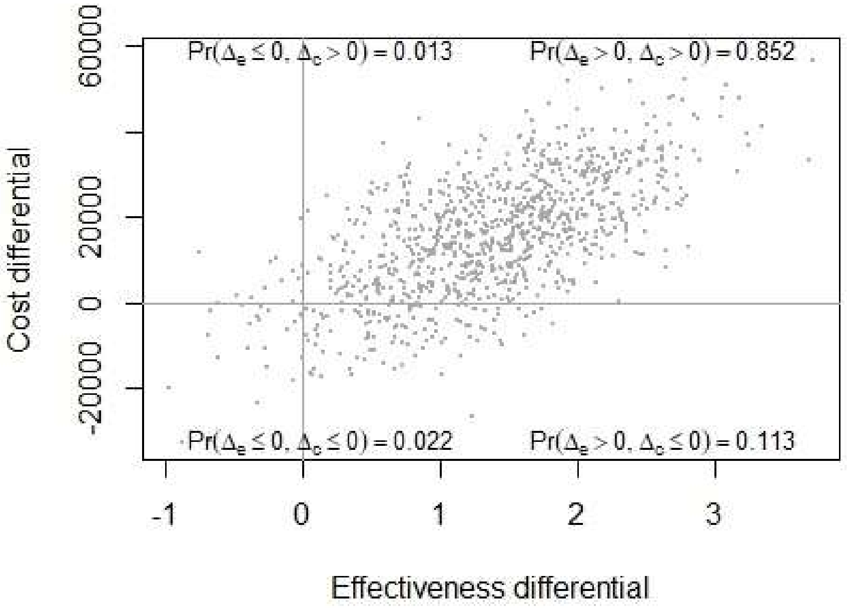

Figure S3. The probabilities that the incremental cost-effect pairs of the MRI screening pathway, relative to the classical screening pathway, to fall in each four quadrants of the cost-effectiveness plain.
