# Supplementary material for: Cost‐effectiveness of multiparametric magnetic resonance imaging and MRI‐guided biopsy in a population‐based prostate cancer screening setting using a micro‐simulation model
Source: Cancer Med. 2021 May 15;10(12):4046–53. doi: 10.1002/cam4.3932 (PMC8209626; doi:10.1002/cam4.3932)
Supplement: Supplementary file 4 — Table S1 [file CAM4-10-4046-s002.docx]

**Supplementary Table 1.** Results of varying the utility estimates and unit costs using a one way sensitivity analysis (each outcome is based on a single run).

|  | | |  |
| --- | --- | --- | --- |
|  | **ICER^*^ in €** |  |  |
| **Utilities** | **Base case** **Favorable^#^**  (=11,135) | **Unfavorable^#^** | |
|  |  |  | |
| utility for screening attendance | Base 11,125 | 11,135 | |
| utility for biopsy | Base 12,970 | 10,067 | |
| utility for diagnosis | Base 11,366 | 10,914 | |
| utility at 2 months after RP treatment | Base 11,870 | 10,815 | |
| utility at 2 months after RT treatment | Base 11,951 | 11,052 | |
| utility at 2 months to 1 year after RP treatment | Base 13721 | 10,177 | |
| utility at 2 months to 1 year after RT treatment | Base 13,741 | 10,105 | |
| utility for AS | Base 12,910 | 10,490 | |
| utility for post recovery period | Base 13,042 | 10,215 | |
| utility for Palliative therapy | Base 12,343 | 10,664 | |
| utility for terminal illness | Base 12,569 | 10,087 | |
| **Costs** | **+15%** | **-15%** | |
| Costs of PSA test | Base 11,164 | 11,107 | |
| Costs of staging | Base 10,852 | 11,253 | |
| Costs of RP | Base 10,658 | 11,530 | |
| Costs of RT | Base 10,476 | 11,794 | |
| Costs of AS | Base 10,019 | 12,253 | |
| Costs of follow-up | Base 10,016 | 11,255 | |
| Costs of advanced case | Base 11,086 | 11,267 | |

| AS = active surveillance, ICER= incremental cost-effectiveness ratio, RP = radical prostatectomy; RT= radiation therapy # the utility estimates are varied using the favorable and unfavorable values based on literature^14^ |
| --- |

^*^The incremental cost effectiveness ratio of the MRI screening pathway as compared to the regular pathway
