# Supplementary material for: Cost‐effectiveness of multiparametric magnetic resonance imaging and MRI‐guided biopsy in a population‐based prostate cancer screening setting using a micro‐simulation model
Source: Cancer Med. 2021 May 15;10(12):4046–53. doi: 10.1002/cam4.3932 (PMC8209626; doi:10.1002/cam4.3932)
Supplement: Supplementary file 1 — Figure S1 [file CAM4-10-4046-s003.pdf]

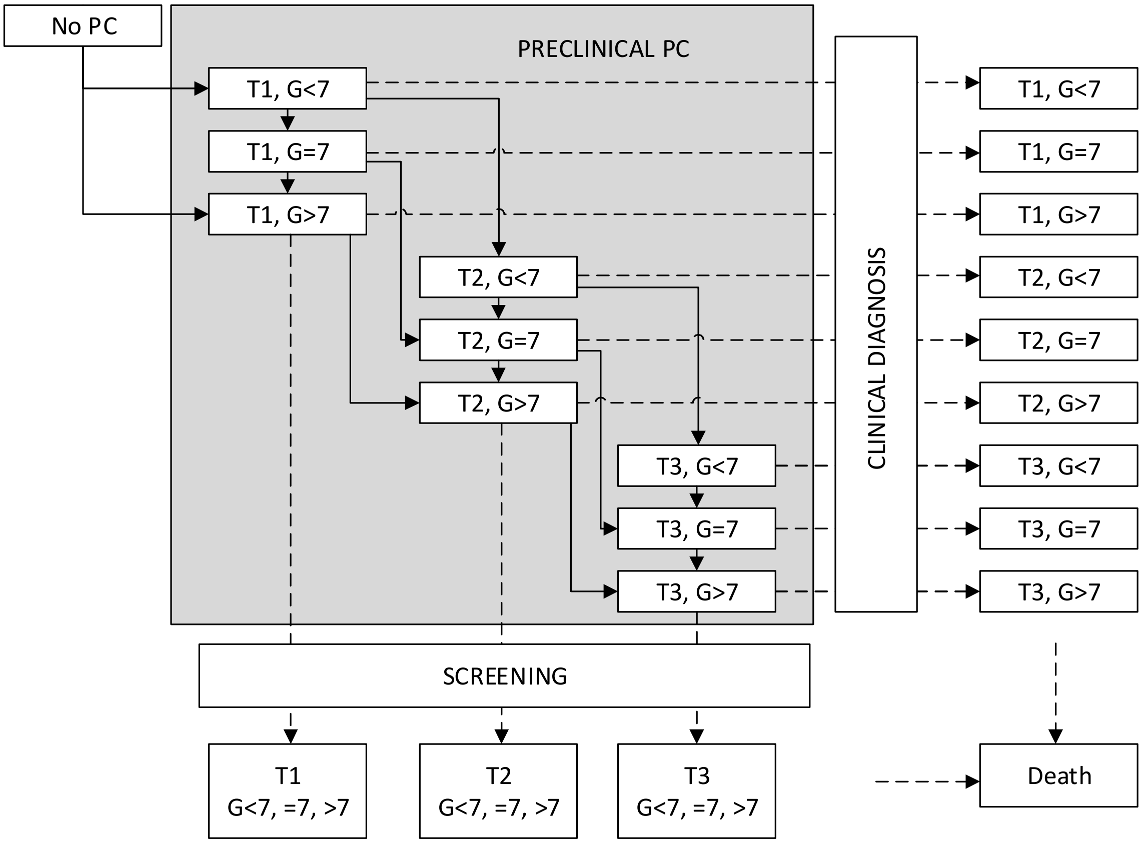

Figure S1. Progression/transition of prostate cancer from no prostate cancer to different preclinical and clinical states. Each states in the model can also be local or distant, but for simplicity this is not included in the figure. T stands for tumor stage and G for Gleason score.
